# Positioning Incretin‐Based and Next‐Generation Obesity Management Medications: A Methodological Framework for a Series of Systematic Reviews and Network Meta‐Analyses

**DOI:** 10.1111/dom.71046

**Published:** 2026-07-01

**Authors:** Matteo Monami, Benedetta Ragghianti, Edoardo Mannucci

**Affiliations:** ^1^ Careggi Teaching Hospital and University of Florence Florence Italy

**Keywords:** incretin, network meta‐analysis, obesity, obesity management medications, pharmacotherapy

## Abstract

**Background:**

The obesity pharmacotherapy landscape is evolving rapidly, with several approved incretin‐based therapies and an expanding pipeline of investigational compounds targeting multiple metabolic pathways. Conventional evidence syntheses often struggle to accommodate differences in dose selection, treatment duration and stage of clinical development.

To describe a methodological framework for a series of systematic reviews and network meta‐analyses aimed at comparing approved and investigational obesity management medications (OMMs).

**Methods:**

This article presents the methodological framework for a series of systematic reviews and network meta‐analyses that will be conducted according to PRISMA 2020, PRISMA‐NMA and PRISMA‐P recommendations. Separate reviews and network meta‐analyses will be performed for individual pharmacological classes of OMMs. Treatment nodes will be defined as molecule‐dose combinations, selecting the highest approved dose for marketed therapies and the highest tested dose for investigational agents. Eligible studies will include randomised Phase 1–4 trials conducted in adults with overweight or obesity, with or without Type 2 diabetes. The primary outcome will be percentage total body weight loss at approximately 24–26 weeks. Risk of bias and certainty of evidence will be assessed using RoB 2 and CINeMA.

**Results:**

The framework identifies 40 obesity investigational molecules that have completed at least Phase 1 development and groups them into 13 major mechanistic classes and nine approved therapies for the treatment of obesity. The present framework, therefore, encompasses 49 molecules which will serve as candidate interventions for future class‐specific network meta‐analyses.

**Conclusions:**

This paper presents a dose‐informed methodological framework for future evidence syntheses in obesity pharmacotherapy, supporting comparative evaluation and contextual positioning of next‐generation OMMs.

**Trail Registration:** PROSPERO—CRD420261425340.

## 1 Introduction

### 1.1 Description of the Condition

Obesity is a major global health challenge associated with increased mortality, multiple chronic diseases, impaired quality of life and substantial economic costs. The therapeutic landscape is rapidly evolving, with an expanding number of pharmacological options demonstrating clinically meaningful effects on body weight. However, important uncertainties remain regarding the comparative efficacy and safety of available and emerging treatments, underscoring the need for comprehensive evidence synthesis [1, 2, 3, 4, 5, 6].

### 1.2 Description of the Intervention and Aims

Weight loss achieved with Glucagon‐like peptide‐1 receptor agonists (GLP‐1 RA), such as semaglutide, and dual GLP‐1/Glucose‐dependent Insulinotropic Peptide receptor agonists (GLP‐1/GIP RA), such as tirzepatide, had been previously reported only with bariatric and metabolic surgery [2, 3]. In the STEP programme, subcutaneous semaglutide 2.4 mg achieved a mean weight reduction of 14.9% at 68 weeks (STEP 1) and 15.2% at 104 weeks (STEP 5) [7]. In the SURMOUNT‐1 trial, tirzepatide 15 mg produced mean weight loss of 20.9% at 72 weeks, with sustained reductions of up to 19.7% at 176 weeks in participants with obesity and prediabetes [8]. The SURMOUNT‐5 head‐to‐head trial confirmed the superiority of tirzepatide over semaglutide, with weight reductions of 20.2% versus 13.7% at 72 weeks [9]. At the same time, a growing number of compounds are progressing through clinical development and are expected to further expand the therapeutic options available. These include oral non‐peptide GLP‐1 RA such as orforglipron, which recently received FDA approval and other peptide and non‐peptide GLP‐1 RA; further GLP‐1/GIP RA beyond tirzepatide; the GLP‐1 agonist/GIP antagonist maridebart cafraglutide; dual GLP‐1/glucagon receptor agonists such as mazdutide (approved in China) and survodutide; the triple GIP/GLP‐1/glucagon receptor agonist retatrutide; several amylin agonists, one of which (cagrilintide) developed also as a fixed‐dose combination with semaglutide; and dual GLP‐1/amylin receptor agonists. Multi‐receptor agonists and novel oral formulations represent the next frontier in obesity pharmacotherapy [10, 11, 12, 13, 14, 15, 16, 17, 18].

This rapid expansion of pharmacological strategies introduces a relevant methodological challenge. Evidence is generated across trials that differ substantially in design features, duration, dose selection and study populations, often preventing direct comparisons [19]. Traditional pairwise meta‐analyses are not suited to address this level of complexity, while conventional network meta‐analyses often rely on simplifying assumptions, such as grouping treatments by class or selecting a single representative dose, potentially masking important pharmacodynamic differences [19, 20]. Recent scoping reviews have highlighted that existing network meta‐analyses of GLP‐1 receptor agonists for weight loss are inconsistent in quality and scope, with a tendency to combine multiple doses and merge findings from multiple time points, limiting understanding of dose and time effects [19, 20]. A meta‐research study of 139 network meta‐analyses found that only 71% of modified PRISMA‐NMA criteria were fulfilled, with network geometry reported in only 15% of cases [20].

### 1.3 Why a New Evidence Synthesis Is Needed

Several systematic reviews, network meta‐analyses and ongoing review protocols have recently addressed pharmacological treatments for obesity [21, 22, 23, 24, 25]. However, most available reviews focus on approved medications, selected incretin‐based therapies or specific clinical settings, whereas the therapeutic landscape is evolving at an unprecedented pace. In the last few months, multiple novel compounds with distinct mechanisms of action, including oral GLP‐1 receptor agonists, GLP‐1/glucagon co‐agonists, GLP‐1/GIP antagonist combinations, amylin‐based therapies and triple agonists, have entered advanced clinical development (Phase 1–3 trials). Existing protocols were generally conceived before much of this evidence became available and therefore cannot fully capture the current therapeutic pipeline. Furthermore, previous network meta‐analyses frequently combined different doses of the same molecule and focused primarily on approved agents (21–25). The present project was specifically designed to address these limitations through a dose‐informed framework, the inclusion of investigational compounds and a comprehensive assessment of emerging obesity pharmacotherapies across stages of development. Therefore, the aim of this review is not to undertake an unnecessary replication of existing evidence syntheses, as cautioned by several authors [26], but to address clinically and methodologically relevant gaps that remain insufficiently covered by previous and ongoing reviews, while providing an updated framework reflecting the rapidly evolving field of obesity pharmacotherapy.

Recent efforts to systematise the comparative evaluation of OMMs have been undertaken at both the European and national level. The European Association for the Study of Obesity (EASO) has developed a GRADE‐based framework for the pharmacological treatment of obesity, using the PICO methodology to formulate clinical questions and planning meta‐analyses and network meta‐analyses to compare EMA‐approved OMMs across patient subgroups [1, 2]. The accompanying systematic review and meta‐analysis, conducted within the EASO framework, evaluated 56 clinical trials enrolling 60 307 patients and demonstrated that all approved OMMs produced a significant weight loss compared to placebo, with semaglutide and tirzepatide achieving reductions over 10% [2]. At the national level, a network meta‐analysis conducted for the development of the SIO (Società Italiana Obesità) Italian guidelines compared EMA‐approved pharmacological, endoscopic and surgical treatments across different BMI‐based classes of obesity, showing that in patients with class I obesity, tirzepatide was equally effective as OAGB and RYGB, while in higher classes of obesity metabolic bariatric surgery was still more effective than OMMs [3]. These analyses, however, were restricted to approved therapies and did not incorporate investigational agents or dose‐level treatment definitions.

### 1.4 Aims

To overcome these limitations, we propose a dose‐informed framework for systematic reviews and network meta‐analyses that explicitly considers dose as an integral component of treatment definition and integrates both approved and investigational therapies within a single analytical structure. The purpose of this approach is not only to compare currently available treatments but also to provide an evidence‐based framework for contextualising emerging agents according to their pharmacological characteristics and observed efficacy profiles. The narrative review of mechanisms of action, the collection of all available ongoing and completed trials, the assessment of efficacy at different time points, and the systematic retrieval of all evidence on adverse events is intended to provide a more definite picture of expected efficacy, safety and tolerability of new agents in comparison with approved drugs.

## 2 Methods

### 2.1 Protocol Description

This article presents an overarching methodological framework for a series of systematic reviews and network meta‐analyses (NMAs), each addressing a specific class of obesity management medications (OMMs; see below). The framework is intended to define common methodological principles and treatment definitions while providing a structured overview of approved and investigational compounds.

The protocol of systematic reviews and NMAs has been prospectively registered in PROSPERO (CRD420261425340; https://www.crd.york.ac.uk/PROSPERO/view/CRD420261425340). Review‐specific protocols provide detailed search strategies, eligibility criteria, outcomes and statistical specifications tailored to each pharmacological class.

Following the PICO framework as suggested by the Preferred reporting items for systematic review and meta‐analysis protocols (PRISMA‐P) checklist (Table S1) [27], the primary review question was: among adults with overweight or obesity (P), what are the comparative efficacy and safety profiles of approved and investigational obesity management medications (I), compared with placebo, standard care, or other obesity medications (C), with respect to weight‐loss efficacy, safety, tolerability and treatment discontinuation outcomes (O)? In addition to the quantitative analysis, these reviews aim at providing a structured and updated overview of pharmacological classes and individual molecules that are currently approved, in advanced clinical development, or approaching regulatory evaluation for the treatment of obesity thereby contextualising the findings within the evolving therapeutic landscape.

For investigational agents, additional information regarding the mechanism of action, sponsor, route of administration, frequency of dosing and stage of clinical development will also be collected whenever available.

### 2.2 Framework‐Level Search Strategy

A preliminary framework‐level search was conducted to identify approved and investigational obesity management medications and to characterise the evolving obesity pharmacotherapy landscape. The purpose of this search was to identify candidate molecules and define the boundaries of future evidence syntheses, rather than to retrieve all randomised trials for quantitative analysis.

Search strategies up to 1 May 2026 are reported in Table S2. Table S2 reported all sources explored, including PubMed, CENTRAL, EMBASE, ClinicalTrials.gov, company pipeline disclosures, corporate websites, conference communications, press releases and grey literature. Information retrieved from different sources was cross‐checked whenever possible.

Because individual systematic reviews and network meta‐analyses will be performed at different time points, review‐specific searches will be updated before study selection. Newly emerging compounds completing at least phase 1 clinical development since the original framework search will be evaluated for inclusion according to predefined eligibility criteria.

Since the planned reviews will be conducted at different time points, review‐specific searches will be updated before study selection to capture newly published evidence and newly emerging compounds. Molecules that have completed at least phase 1 clinical development since the original framework search will be considered for inclusion according to the predefined eligibility criteria. This dynamic approach is intended to preserve both prospective registration and the currency of the evidence base in a rapidly evolving field.

### 2.3 Types of Studies and Participants

The methodological approach builds upon the analytical framework previously developed for the EASO GRADE‐based guidelines and the SIO Italian guidelines (we will perform an update up to May, 2026, also including shorter‐term trials, previously excluded), extending it to incorporate investigational agents and dose‐level treatment definitions [1, 3]. Eligible studies will include Phase 1–4 randomised trials conducted in adult populations with overweight or obesity (either BMI ≥ 27 kg/m^2^ with at least one obesity‐associated comorbid condition or BMI ≥ 30 kg/m^2^), with or without Type 2 diabetes. While a minimum trial duration of 24 weeks will be required for inclusion in the primary analysis to ensure adequate characterisation of weight loss trajectories, shorter‐duration randomised trials (≥ 12 weeks) will be included in sensitivity analyses. This approach was adopted to provide preliminary insights into the efficacy of early‐phase compounds and to enhance the representation of emerging therapies within the network, while preserving the robustness of the primary estimates [19, 20]. Only published English‐language studies will be eligible for inclusion. Studies conducted exclusively in paediatric populations. Non‐randomised studies will be excluded, as well. We will not exclude studies based on outcome reporting. Studies reporting none of the predefined efficacy or safety outcomes will be retained for descriptive purposes only.

### 2.4 Types of Intervention and Comparator

Interventions were classified according to their primary mechanism of action into major pharmacological groups, that is, GLP‐1 receptor agonists, oral GLP‐1 small molecules, dual GLP‐1/GIP agonists, dual GLP‐1/glucagon agonists, GLP‐1 agonist/GIP antagonist combinations, triple GIP/GLP‐1/glucagon agonists, GLP‐1/amylin combinations, etc. Within each class, individual molecules will be described in terms of their mechanism of action, route of administration and stage of clinical developments. Older drugs approved for obesity (e.g., orlistat, naltrexone/buproprione, phentermine/topiramate, Table 1) were excluded from the analysis because of their reported lower efficacy on weight loss when compared to incretin‐based drugs [2, 3].

**TABLE 1:** Oral and injectable Glucagon‐Like Peptide‐1 Receptor Agonists (GLP‐1 RAs) in development for the treatment of obesity (as of 1 May 2026).

Drug	Code	Adm.	Route	Company	Clinical phase
Injectable					
Berobenatide	PF‐08653944/MET‐097	OM	SC	Pfizer/Metsera	In development
Exenatide implant	NPM‐115/NPM‐119	6M	SC	Vivani Medical	In development
Bofanglutide	—	OW	SC	Gan&Lee Pharmaceuticals/Carnot	In development
Oral					
Elecoglipron	AZD5004/ECC‐5004	OD	Oral	AstraZeneca/Eccogene	In development
Aleniglipron	GSBR‐1290	OD	Oral	Gasherbrum Bio	In development
Tifeglipron	CT‐996/RG‐6652	OD	Oral	Roche/Carnot/Hoffmann	In development
Safiglipron	KAI‐7535/HRS‐7535	OD	Oral	Shandong Suncadia Medicine/Kailera	In development
TERN‐601	TERN‐601	OD	Oral	Merck/Terns Pharma	In development
RGT‐075	RGT‐075	OD	Oral	Regor Therapeutics	In development
AZD9550	AZD9550	OD	Oral	AstraZeneca	In development
Danuglipron	PF‐06882961	BD	Oral	Pfizer	Suspended
Lotiglipron	PF‐07081532	OD	Oral	Pfizer	Discontinued

**TABLE 2:** Multiple agonist‐obesity management medications in development for the treatment of obesity (as of 1 April 2026).

Drug	Code	Adm.	Route	Company	Clinical phase
Dual GLP‐1/GIP agonists					
Enicepatide	CT‐388; RG‐6640; RO‐7795068	OW	SC	Roche/Carmot	In development
Olatorepatide	HS‐20094	OW	SC	Jiangsu Hansoh	In development
Acmopatide	CT‐868/RG‐6641	OW	SC	Roche/Carmot	In development
Ribupatide	HRS 9531/KAI‐9531	OW	SC	Kailera/Fujian Shengdi	In development
VK‐2735	VK‐2735	OW	SC and oral	Viking	In development
Dual GLP‐1/glucagon agonists					
Survodutide	BI456906	OW	SC	Boehringer Ingelheim	In development
Pemvidutide	ALT‐801/SP‐1373; VPD‐107	OW	SC	Altimmune/Velocity	In development
Efinopegdutide	HM‐12525A; JNJ 5111	OW	SC	Hanmi/Janssen	In development
GLP‐1 agonist + GIP antagonist					
Maridebart cafraglutide	AMG133/Maritide	OM	SC	Amgen	In development
Triple agonists (GLP‐1/GIP/glucagon)					
Retatrutide	LY‐3437943	OW	SC	Eli Lilly	In development
Efocipegtrutide	HM15211	OW	SC	Hanmi	In development
NN‐9559	UBT251	OW	SC	Novo Nordisk/United Biotechnology	In development
SAR‐441255	SAR‐441255	OW	SC	Sanofi	Discontinued
Triple agonists (GLP‐1/GIP/amylin)					
NN triple	—	OW	SC	Novo Nordisk	In development
Quadruple agonists (GLP‐1/GIP/glucagon ± IGF‐1 axis)					
BioGlutide	NA‐931	OD	Oral	Biomed Industries	In development
Dual GLP‐1 + amylin agonist					
Cagrilintide/semaglutide	CagriSema; NN‐9388	OW	SC	Novo Nordisk	In development
Zenagamtide	Amycretin/NN9487, NNC0487‐0111	OD	Oral	Novo Nordisk	In development
Zenagamtide	Amycretin/NN9487, NNC0487‐0111	OW	SC	Novo Nordisk	In development
Dual GLP‐1/GLP‐2 receptor agonist					
Dapiglutide	ZP7570	OW	SC	Zealand Pharma	Suspended
Bimagrumab	LY‐3985863/BYM‐338	OM	IV/SC	Eli Lilly/Novartis	In development

Abbreviations: 6M, every 6 months; BD, twice daily; OD, once daily; OM, once monthly; OW, once weekly; SC, subcutaneous.

Established approved therapies for obesity (i.e., liraglutide, s.c. semaglutide, oral semaglutide, ecnoglutide, tirzepatide, orforglipron and mazdutide) will be considered alongside investigational compounds at the maximum (approved or tested) dose. The descriptive synthesis will not be restricted to agents included in the quantitative analysis but will also encompass emerging therapies for which evidence may still be limited, thereby providing a forward‐looking perspective. When multiple doses of the same intervention are evaluated within a trial, only the treatment arm corresponding to the predefined dose‐selection strategy will contribute to the primary network meta‐analysis. Information from alternative doses will be collected and may be reported descriptively or incorporated into supplementary analyses when appropriate.

The comparator will be lifestyle modification with either a placebo or an alternative active drug. Trials incorporating background co‐interventions will be considered eligible provided that such interventions are applied equally across all randomised groups, thereby preserving the validity of treatment comparisons. In multi‐arm studies, all treatment groups meeting the predefined eligibility criteria will be considered for inclusion in the evidence synthesis.

### 2.5 Types of Outcome Measures

#### 2.5.1 Primary Outcome

− Percent change in total body weight from baseline (TBWL%) at approximately 24–26 weeks. This timepoint was selected to maximise inclusion of emerging compounds and early‐phase studies, while maintaining clinical relevance.

#### 2.5.2 Secondary Outcomes

− Percent change in total body weight from baseline at longer follow‐up durations (i.e., 52 weeks and ≥ 104 weeks).
− Categorical weight‐loss targets, including the proportion of participants achieving at least 5%, 10%, 15%, 20% and 25% weight reduction from baseline.
− Health‐related quality of life, assessed using validated instruments whenever available.
− Tolerability outcomes, including gastrointestinal adverse events and treatment discontinuation due to adverse events.
− Safety outcomes, including all‐cause mortality, major adverse cardiovascular events (MACE), serious adverse events (SAEs), discontinuation due to SAEs and discontinuation for either gastrointestinal problems or any reason.
− Additional safety outcomes of particular relevance to obesity pharmacotherapy, including pancreatitis, gallbladder and liver disease, psychiatric adverse events and changes in body composition (including lean mass) when reported.

When multiple outcome assessments are available within a given study, the timepoint closest to the prespecified follow‐up window will be selected for analysis.

### 2.6 Data Collection, Extraction and Analysis

Data collection and extraction (independently performed by two authors: M.M. and B.R.) will be performed at the level of individual trial arms and will include sample size, baseline characteristics (e.g., first author, year of publication, mean age, BMI, HbA1c, proportion of enrolled women, etc.), intervention details and outcomes (see above), as assessed by intention‐to‐treat analyses or treatment policy estimands. We will resolve disagreements through consensus.

### 2.7 Assessment of Risk of Bias in Included Studies

Two review authors (M.M., B.R.) will independently assess the risk of bias in each study using the Cochrane Risk of Bias 2 (RoB 2) tool [28] and disagreements will be resolved by consensus.

### 2.8 Measures of Treatments Effects

For binary outcomes, treatment effects will be summarised using risk ratios (RRs) with corresponding 95% confidence intervals (CIs). Continuous outcomes assessed using a common measurement scale will be analysed as mean differences (MDs) with 95% CIs, whereas outcomes measured using different instruments will be synthesised using standardised mean differences (SMDs) with 95% CIs. When appropriate, SMDs may be converted into more clinically interpretable metrics using representative standard deviations derived from the included studies. Time‐to‐event outcomes will be analysed using hazard ratios (HRs) and associated 95% CIs. Interventions will be ranked using established ranking metrics for network meta‐analysis. Ranking probabilities and summary ranking measures, including the Surface Under the Cumulative Ranking Curve (SUCRA), may be calculated to facilitate interpretation of the relative performance of competing treatments. SUCRA values range from 0 to 1, with higher values indicating a greater probability of occupying more favourable positions within the treatment hierarchy. Detailed analytical specifications, including software implementation and ranking procedures, will be reported in the corresponding review protocols.

### 2.9 Management of Missing Data

Whenever relevant information is missing or unclear, additional sources (e.g., supplementary appendices, trial registries, protocols, regulatory documents or author correspondence) will be consulted. Any data derivation or imputation procedures will be transparently reported and justified in the corresponding review. Missing data and the methods used by the original investigators to handle them will be documented during data extraction and considered in risk‐of‐bias assessments. Where appropriate, standard imputation methods may be applied, and the impact of any imputed data will be explored through sensitivity analyses. All assumptions and imputation procedures will be transparently reported in the corresponding review publications.

### 2.10 Assessment of Heterogeneity, Transitivity and Inconsistency

Clinical and methodological heterogeneity will be evaluated by examining study design, participant characteristics, intervention features and outcome definitions across included studies. Particular attention will be given to potential effect modifiers, including baseline BMI, diabetes status, study duration and trial phase. Between‐study heterogeneity will be quantified using standard metrics (*I* ^2^ and *τ* ^2^) and interpreted in conjunction with clinical and methodological considerations [19, 20, 28]. Whenever sufficient studies are available, small‐study effects and publication bias will be explored using graphical methods (including funnel plot inspection) and, where appropriate, statistical approaches (e.g., Egger's test). Potential meta‐biases will be assessed whenever feasible, depending on the number of available studies within each network.

The assumptions of transitivity and network coherence will be formally evaluated before conducting network meta‐analyses.

Consistency between direct and indirect evidence will be evaluated using established global and local approaches for network meta‐analysis. Where inconsistency is detected, its potential sources will be explored through additional analyses, and the implications for interpretation will be carefully considered. In the presence of disconnected networks, interventions may be analysed within separate subnetworks, described narratively or incorporated through model‐based approaches when appropriate assumptions are met.

Where substantial heterogeneity, inconsistency or lack of transitivity is identified, results will be interpreted cautiously, and quantitative synthesis may be restricted or replaced by a narrative summary [19, 20].

### 2.11 Assessment of Reporting Bias

Potential reporting biases, including publication bias and small‐study effects, will be assessed whenever sufficient data are available. Graphical and statistical approaches appropriate for pairwise and network meta‐analysis may be applied, and any evidence of reporting bias will be considered during interpretation of the findings and assessment of evidence certainty [28, 29].

### 2.12 Certainty of Evidence

The certainty of evidence for each outcome and treatment comparison will be evaluated using the CINeMA (Confidence in Network Meta‐Analysis) framework, which considers six domains: within‐study bias, reporting bias, indirectness, imprecision, heterogeneity and incoherence [28, 29]. The overall certainty of evidence will be rated as high, moderate, low or very low according to judgements across these domains.

### 2.13 Network Structure and Treatment Definitions

Whenever sufficient evidence is available, separate network meta‐analyses (NMAs) will be performed for each pharmacological class of obesity management medications (OMMs), including both approved and investigational compounds. Eligible studies will include Phase 1–4 randomised controlled trials conducted in adults with overweight or obesity, with or without Type 2 diabetes.

Treatment nodes will be defined as molecule‐dose combinations. For approved obesity medications, the highest approved dose will be selected to represent the maximal clinically available treatment effect (e.g., liraglutide 3.0 mg once daily, subcutaneous semaglutide 2.4 mg once weekly, oral semaglutide 50 mg once daily, tirzepatide 15 mg once weekly, orforglipron 36 mg once daily, mazdutide 6 mg once weekly and ecnoglutide 2.4 mg once weekly). For investigational compounds, the highest dose evaluated within randomised clinical trials will be selected to approximate maximal observed pharmacodynamic efficacy. Although such doses may not correspond to those ultimately adopted in clinical practice, this approach was chosen to provide a consistent framework for comparing approved and emerging therapies. Consequently, treatment‐effect estimates should be interpreted as reflecting maximal observed efficacy under trial conditions rather than the expected effectiveness of finalised therapeutic regimens.

Each molecule‐dose combination will contribute as a distinct node within the network. Placebo will serve as the reference treatment whenever available, allowing estimation of both direct and indirect treatment effects across the network. Doses other than the predefined maximal dose will not be included in the primary NMA but will be systematically collected and may be explored through supplementary analyses to characterise dose–response relationships within individual molecules.

Network meta‐analysis will only be undertaken when assumptions of clinical similarity, transitivity and network coherence are considered sufficiently plausible. However, to explore potential violations of the transitivity assumption, meta‐regression analyses may be performed using relevant study‐level covariates, including trial duration, age, ethnicity, diabetes status and baseline body mass index, whenever sufficient data are available [19, 20]. Analyses will be stratified according to the presence or absence of Type 2 diabetes, given the known differences in treatment response between these populations [4, 22, 23]. Sensitivity analyses may include restriction to Phase 3 trials, exclusion of studies at high risk of bias, and evaluation of longer‐duration studies to assess the robustness of the findings. Phase 2 studies could yield different efficacy results compared to Phase 3 trials, even at corresponding time points (i.e., 24–26 weeks), due to differences in case mix, choice of investigational centres or study procedures. For this reason, a sensitivity NMA will be performed including only Phase 2 studies for all molecules. Additional sensitivity analyses may be performed excluding studies requiring imputation of standard deviations or other variance measures, whenever sufficient data are available.

Network meta‐analyses will be conducted using random‐effects models implemented through MetaInsight (version 6.0.0 or later), a web‐based platform for evidence synthesis and network meta‐analysis. Treatment effects will be estimated relative to placebo and, where available, relative to active comparators. Data extraction will be performed at the level of individual trial arms and will include sample size, baseline characteristics, intervention details and outcome data derived from intention‐to‐treat analyses or treatment‐policy estimands whenever available [19, 20, 28].

## 3 Results

Earlier anti‐obesity agents, including phentermine (alone or associated with topiramate), mazindole, bupropion/naltrexone and orlistat, are summarised separately (Table S3) as they represent a previous generation of pharmacological approaches. These compounds act primarily through central appetite modulation or peripheral inhibition of nutrient absorption and are characterised by modest efficacy and less favourable tolerability profiles compared with newer therapies. In clinical practice, their use has progressively declined with the advent of incretin‐based treatments, which provide substantially greater and more sustained weight reduction. Accordingly, these agents will not be included in the primary comparative analyses of the present study, which will focus on contemporary incretin‐based pharmacotherapies. Similarly, the melanocortin‐4 agonist setmelanotide, although approved for use in the United States (US) and in the United Kingdom (UK), will not be included in comparisons with newer drugs, since its use is limited to some forms of monogenic obesity, and it is not intended for the treatment of most cases of obesity.

### 3.1 Approved Incretin‐Based OMM and Newer Investigational Agents

Nine agents have been approved for the treatment of obesity in either Europe, the UK, the US, China, Japan, India or Australia (Table S4).

Tables 1, 2, 3 include 40 pharmacological agents for obesity that have completed at least Phase 1 clinical trials. Information regarding mechanisms of action, stage of development, sponsoring companies and available clinical evidence was retrieved and cross‐checked across sources whenever possible. Data on completed, ongoing and terminated clinical trials were collected to provide a descriptive overview of the evidence landscape. These compounds can be grouped in 13 mechanistic classes; most compounds, accounting for roughly 70% of the pipeline, target the incretin axis, either as GLP‐1 receptor agonists or as multi‐agonist combinations (Tables 1, 2, 3). Oral agents represent approximately one‐third of all candidates, largely driven by the expansion of non‐peptide GLP‐1 receptor agonists (Table 1).

**TABLE 3:** Other non‐incretin‐based classes of drugs in development for the treatment of obesity (as of 1 April 2026).

Drug	Code	Adm.	Route	Company	Clinical phase
Amylin receptor agonists					
Cagrilintide	NN9838/AM‐833	OW	SC	Novo Nordisk	In development
Eloralintide	LY‐3841136	OW	SC	Eli Lilly	In development
Petrelintide	RG‐6849/ZP‐8396	OW	SC	Roche/Zealand	In development
PF‐08653945	MET‐233	OW	SC and oral	Pfizer/D and D Pharmatech	In development
AZD‐6234	AZD‐6234	OW	SC	AstraZeneca	In development
AT‐7687	AT‐7687	OW	SC	Antag Therapeutics	In development
Cannabinoid receptor blocker‐1 (inverse agonist)					
Monlunabant	INV‐202	OD	Oral	Novo Nordisk/Inversago	In development
Activin Type II receptor blockade (muscle‐targeting)					
Bimagrumab	BYM338	OM	IV/SC	Eli Lilly	In development

Abbreviations: 6M, every 6 months; BD, twice daily; CB1, cannabinoid receptor Type 1; IV, intravenous; OD, once daily; OM, once monthly; OW, once weekly; SC, subcutaneous.

Therefore, the present framework encompasses 49 molecules (Tables 1, 2, 3 and Table S4), including 9 approved OMMs and 40 investigational agents or novel doses of approved therapies, which will serve as candidate interventions for future class‐specific network meta‐analyses.

### 3.2 Mechanistic Landscape

Incretin‐based therapies constitute the dominant class within the current pipeline, encompassing injectable and oral GLP‐1 receptor agonists as well as dual and triple agonists engaging GLP‐1, GIP and glucagon receptors. These agents primarily act through central appetite suppression, delayed gastric emptying and modulation of insulin–glucagon dynamics, with additional contributions from increased energy expenditure in glucagon‐containing co‐agonists. Over time, the field has evolved from peptide full agonists towards more complex pharmacological strategies, including biassed agonism among small molecules and multi‐receptor targeting designed to enhance efficacy.

Multi‐agonist approaches [12, 14, 16, 30, 31, 32, 33] represent a substantial proportion of compounds in mid‐ to late‐stage development. By simultaneously activating complementary metabolic pathways, these agents aim to reproduce and amplify physiological nutrient signaling. Dual GLP‐1/GIP agonism improves insulin sensitivity and adipose tissue metabolism, while GLP‐1/glucagon agonism introduces an energy expenditure component through increased lipid oxidation. In addition, the stimulation of glucagon receptor has a relevant effect on hepatic lipid metabolism, leading to a substantial clinical improvement of Metabolic dysfunction‐Associated Steatotic Liver Disease (MASLD) [32], to a possibly greater extent than GLP‐1 RA and GLP‐1/GIP RA [30]. Triple agonists integrate these mechanisms and have demonstrated marked weight reductions in early clinical studies [12].

Amylin‐based therapies form a distinct and increasingly relevant class, acting through central satiety pathways that are complementary to GLP‐1 signalling. This biological rationale supports combination strategies, particularly GLP‐1 plus amylin approaches, which aim to enhance weight loss while potentially improving tolerability through dose optimisation [31]. Alternatively, both GLP‐1 and amylin receptors can be activated by using a co‐agonist [31].

A smaller but mechanistically diverse group of agents targets non‐incretin pathways. These include peripheral cannabinoid receptor Type 1 inverse agonists [33], designed to modulate appetite and metabolism without central nervous system adverse effects, and activin Type II receptor blockade [34], which aims to preserve or increase lean mass and thereby improve body composition. These approaches reflect an emerging focus on the qualitative aspects of weight loss rather than weight reduction alone.

## 4 Discussion

The evaluation and comparative positioning of novel obesity management medications, particularly those belonging to emerging pharmacological classes, are intrinsically complex. This complexity is amplified in obesity research by several methodological and clinical factors influencing both efficacy and safety estimates.

Treatment efficacy is strongly time‐dependent. Weight‐loss trajectories differ substantially across pharmacological classes, with more potent agents typically achieving their maximal effect only after prolonged exposure. Consequently, shorter trials, especially early‐phase studies, tend to underestimate the full therapeutic potential of these compounds. This temporal dimension represents a major source of heterogeneity when comparing agents evaluated at different time points.

In addition, the presence of Type 2 diabetes significantly modifies treatment response. Improvements in glycaemic control are known to attenuate weight loss, leading to systematically lower efficacy estimates in diabetic populations compared with non‐diabetic individuals [4, 18]. This issue is particularly relevant in network meta‐analyses, where differences in the proportion of participants with diabetes across trials may violate the transitivity assumption.

Variations in case mix further complicate comparisons. Baseline BMI, sex distribution, ethnicity and geographic origin all influence treatment response. Trials conducted in Asian populations, often characterised by lower baseline BMI, may report smaller absolute weight reductions, potentially leading to misleading cross‐trial comparisons if not appropriately adjusted [16, 35].

Moreover, differences across trial phases introduce additional variability. Phase 2 studies often differ from Phase 3 trials in terms of population selection, trial conduct, dose‐escalation protocols and concomitant interventions. These differences may translate into divergent efficacy estimates even at similar time points. To address this issue, the present protocol includes sensitivity analyses restricted to Phase 2 trials, allowing a more homogeneous comparison of early‐phase data and improving the interpretability of results for investigational compounds.

Similarly, tolerability represents a challenging domain. Gastrointestinal adverse events, typical of incretin‐based therapies, are influenced not only by drug mechanisms but also by titration schedules, trial duration and patient characteristics. Importantly, tolerability cannot be adequately captured by incidence rates alone. The clinical impact depends on symptom severity, duration, reversibility and treatment discontinuation rates. Distinguishing between transient mild symptoms and persistent severe events is therefore essential for a clinically meaningful interpretation of tolerability profiles.

Assessment of safety is even more complex. Most available data for investigational agents derive from relatively short‐duration trials that are not powered to detect rare or long‐term adverse outcomes, including cardiovascular events. As a result, safety evaluation must integrate trial data with mechanistic considerations and emerging evidence from longer‐term studies and post‐marketing surveillance of related compounds.

Finally, study‐design characteristics, including comparator choice, background lifestyle interventions and endpoint definitions, may substantially influence observed outcomes. For this reason, the present framework incorporates all available evidence, including ongoing trials, to provide a comprehensive and forward‐looking assessment of the therapeutic landscape.

Taken together, these considerations underscore the need for a structured and flexible analytical framework. The series of dose‐informed network meta‐analyses proposed in this protocol is specifically designed to address these challenges by standardising treatment definitions, incorporating key sources of heterogeneity through meta‐regression and stratification, and enabling more granular comparison of approved and investigational therapies. Because analyses will be conducted within pharmacological classes, the framework should not be interpreted as generating a universal ranking across all approved and investigational agents. Rather, it is intended to provide class‐specific comparative evidence and to contextualise emerging therapies within the broader obesity treatment landscape.

Several limitations should nevertheless be acknowledged.

Despite the use of meta‐regression and stratified analyses to account for key effect modifiers such as trial duration, baseline BMI and diabetes status, residual heterogeneity and confounding cannot be fully excluded. The assumption of transitivity, which underpins network meta‐analysis, may be challenged by differences in case mix, study design and background interventions across trials.

Moreover, the definition of treatment nodes at the molecule‐dose level, while improving pharmacological resolution, requires the selection of a single dose per agent. For approved therapies, the highest licenced dose reflects clinical practice; however, for investigational compounds, the use of the highest tested dose represents maximal observed efficacy under trial conditions rather than the dose likely to be adopted in real‐world settings. This approach may therefore overestimate comparative efficacy and does not fully capture dose–response relationships, which are addressed only in secondary analyses. The proposed framework should be viewed as a comparison of currently observed maximal efficacy signals rather than a comparison of finalised therapeutic strategies or expected real‐world effectiveness.

In addition, a substantial proportion of evidence for emerging agents derives from Phase 2 trials. These studies are generally smaller, shorter and more heterogeneous than Phase 3 trials, with differences in population selection, titration schemes and study conduct. Consequently, efficacy and tolerability estimates for investigational drugs should be interpreted cautiously, as they may not fully translate into later‐phase or real‐world outcomes.

The temporal dimension of weight loss represents a further limitation. The selection of the 24–26‐week timepoint resulted from extensive discussion among the authors and reflects a deliberate balance between clinical relevance and inclusiveness of emerging evidence. The primary analysis at 24–26 weeks ensures comparability across a larger number of trials but may underestimate the full effect of agents with slower or progressive weight‐loss trajectories. Although longer follow‐up may provide a more complete assessment of therapeutic durability, a later primary timepoint would substantially restrict the inclusion of investigational compounds and early‐phase studies, thereby limiting the framework's ability to capture the evolving obesity pharmacotherapy landscape. However, longer‐term outcomes are included as secondary endpoints and they could provide useful information, despite being in a limited number of studies.

The framework is expected to provide the most reliable comparative evidence for weight‐loss efficacy and, to a lesser extent, treatment discontinuation and tolerability. Conversely, rare or long‐term safety outcomes are likely to remain incompletely characterised, particularly for investigational compounds and early‐phase studies, and should therefore be interpreted cautiously. Tolerability assessments are constrained by heterogeneity in reporting and by the multidimensional nature of adverse events. Gastrointestinal symptoms, which dominate incretin‐based therapies, vary in severity, duration, and clinical relevance, and incidence rates alone may not adequately capture their impact on patients. Discontinuation rates partly address this limitation but remain an imperfect proxy.

Furthermore, safety evaluation is limited by the relatively short duration and sample size of most trials, particularly for investigational agents. Rare or long‐term adverse outcomes, including cardiovascular events, cannot be reliably assessed within this framework and require dedicated outcome trials and post‐marketing data.

In addition, the geographic and demographic composition of available evidence may limit generalisability. Most trials have been conducted predominantly in Caucasian populations, with notable contributions from Asian cohorts differing in baseline characteristics such as BMI and body composition. These differences may influence efficacy and tolerability and are only partially captured in the analysis.

Although the inclusion of approved and investigational agents provides a comprehensive and forward‐looking overview, the network remains inherently dynamic. As new trials are completed and additional therapies enter clinical development, estimates may change and the relative positioning of treatments will require continuous updating. Future iterations of this framework may incorporate more advanced methodologies, including model‐based dose–response network meta‐analysis, dose‐effect meta‐regression and hierarchical models integrating dose and pharmacological class information, particularly as the evidence base for emerging compounds becomes more robust.

In conclusion, this protocol introduces a structured, dose‐informed framework for the comparative evaluation and contextual interpretation of OMMs within an increasingly complex and rapidly evolving therapeutic landscape. Importantly, this approach emphasises the need to interpret efficacy alongside tolerability and safety, recognising that the clinical value of OMMs lies in the balance between magnitude of weight loss, prevention and improvement of obesity‐associated complications, treatment acceptability and improvement in quality of life. The planned integration of efficacy‐tolerability profiling further supports a multidimensional assessment aligned with real‐world decision‐making and contemporary obesity‐management frameworks [1, 36, 37, 38, 39].

## Author Contributions

Conceptualisation: Matteo Monami and Edoardo Mannucci. Methodology: Matteo Monami and Edoardo Mannucci. Drafting of the manuscript: Matteo Monami, Edoardo Mannucci and Benedetta Ragghianti. Supervision: Matteo Monami. Guarantor of the study: Matteo Monami. All authors read and approved the final manuscript.

## Funding

The authors have nothing to report.

## Consent

The authors have nothing to report.

## Conflicts of Interest

E.M. received consultancy and/or speaking fees from Eli Lilly, Novo Nordisk and Sanofi, and research grants from AstraZeneca, Eli Lilly, Genentech, Novo Nordisk and Sanofi. M.M. received speaking fees from Eli Lilly and Novo Nordisk. B.R. has no conflicts of interest to declare.

## Supporting information


**Table S1:** A PRISMA‐P checklist for the assessment of protocol completeness and adherence to current reporting standards for systematic review protocols.
**Table S2:** Search strategies for identifying potential investigational drugs.
**Table S3:** Earlier‐generation anti‐obesity pharmacotherapies with limited contemporary use
**Table S4:** Classes of drugs and individual obesity management medications approved for the treatment of obesity (as of 1 April 2026).

## Data Availability

All data relevant to the study are included in the article or uploaded as Supporting Information.
