# Supplementary material for: Positioning Incretin‐Based and Next‐Generation Obesity Management Medications: A Methodological Framework for a Series of Systematic Reviews and Network Meta‐Analyses
Source: Diabetes Obes Metab. 2026 Jul 1;28(9):7768–78. doi: 10.1111/dom.71046 (PMC13449067; doi:10.1111/dom.71046)
Supplement: Supplementary file 1 [file DOM-28-7768-s001.docx]

**Table 1S** – A PRISMA-P checklist for the assessment of protocol completeness and adherence to current reporting standards for systematic review protocols.

| **Section/topic** | **#** | **Checklist item** | **Information reported** | | **Line number(s)** |
| --- | --- | --- | --- | --- | --- |
|  |  |  | **Yes** | **No** |  |
| **ADMINISTRATIVE INFORMATION** | | | | | |
| **Title** | | | | | |
| Identification | 1a | Identify the report as a protocol of a systematic review | ☒ | ☐ | 2, 34, 137 |
| Update | 1b | If the protocol is for an update of a previous systematic review, identify as such | ☒ | ☐ | NA |
| **Registration** | 2 | If registered, provide the name of the registry (e.g., PROSPERO) and registration number in the Abstract | ☐ | ☒ | Not done |
| **Authors** | | | | | |
| Contact | 3a | Provide name, institutional affiliation, and e-mail address of all protocol authors; provide physical mailing address of corresponding author | ☒ | ☐ | 17-19 |
| Contributions | 3b | Describe contributions of protocol authors and identify the guarantor of the review | ☒ | ☐ | 394-422 |
| **Amendments** | 4 | If the protocol represents an amendment of a previously completed or published protocol, identify as such and list changes; otherwise, state plan for documenting important protocol amendments | ☒ | ☐ | NA |
| **Support** | | | | | |
| Sources | 5a | Indicate sources of financial or other support for the review | ☒ | ☐ | 417-419 |
| Sponsor | 5b | Provide name for the review funder and/or sponsor | ☒ | ☐ | NA |
| Role of sponsor/funder | 5c | Describe roles of funder(s), sponsor(s), and/or institution(s), if any, in developing the protocol | ☒ | ☐ | NA |
| **INTRODUCTION** | | | | | |
| **Rationale** | 6 | Describe the rationale for the review in the context of what is already known | ☒ | ☐ | 100-107; 122-131 |
| **Objectives** | 7 | Provide an explicit statement of the question(s) the review will address with reference to participants, interventions, comparators, and outcomes (PICO) | ☒ | ☐ | 140-145 |
| **METHODS** | | | | | |
| **Eligibility criteria** | 8 | Specify the study characteristics (e.g., PICO, study design, setting, time frame) and report characteristics (e.g., years considered, language, publication status) to be used as criteria for eligibility for the review | ☒ | ☐ | 140-145, 159-171 |
| **Information sources** | 9 | Describe all intended information sources (e.g., electronic databases, contact with study authors, trial registers, or other grey literature sources) with planned dates of coverage | ☒ | ☐ | 154-159 |
| **Search strategy** | 10 | Present draft of search strategy to be used for at least one electronic database, including planned limits, such that it could be repeated | ☒ | ☐ | 154-159; 170-172 |
| ***STUDY RECORDS*** | | | | | |
| Data management | 11a | Describe the mechanism(s) that will be used to manage records and data throughout the review | ☒ | ☐ | 160-165; 185-209 |
| Selection process | 11b | State the process that will be used for selecting studies (e.g., two independent reviewers) through each phase of the review (i.e., screening, eligibility, and inclusion in meta-analysis) | ☒ | ☐ | 216 |
| Data collection process | 11c | Describe planned method of extracting data from reports (e.g., piloting forms, done independently, in duplicate), any processes for obtaining and confirming data from investigators | ☒ | ☐ | 216-226 |
| **Data items** | 12 | List and define all variables for which data will be sought (e.g., PICO items, funding sources), any pre-planned data assumptions and simplifications | ☒ | ☐ | 227-229 |
| **Outcomes and prioritization** | 13 | List and define all outcomes for which data will be sought, including prioritization of main and additional outcomes, with rationale | ☒ | ☐ | 218-226 |
| **Risk of bias in individual studies** | 14 | Describe anticipated methods for assessing risk of bias of individual studies, including whether this will be done at the outcome or study level, or both; state how this information will be used in data synthesis | ☒ | ☐ | 252-255 |
| ***DATA*** | | | | | |
| **Synthesis** | 15a | Describe criteria under which study data will be quantitatively synthesized | ☐ | ☐ |  |
|  | 15b | If data are appropriate for quantitative synthesis, describe planned summary measures, methods of handling data, and methods of combining data from studies, including any planned exploration of consistency (e.g., *I* ^2^, Kendall’s tau) | ☒ | ☐ | 230-236 |
|  | 15c | Describe any proposed additional analyses (e.g., sensitivity or subgroup analyses, meta-regression) | ☒ | ☐ | 239-245 |
|  | 15d | If quantitative synthesis is not appropriate, describe the type of summary planned | ☒ | ☐ | NA |
| **Meta-bias(es)** | 16 | Specify any planned assessment of meta-bias(es) (e.g., publication bias across studies, selective reporting within studies) | ☒ | ☐ | 248-252 |
| **Confidence in cumulative evidence** | 17 | Describe how the strength of the body of evidence will be assessed (e.g., GRADE) | ☒ | ☐ | 253-258 |

NA: Not Applicable

**Table 2S –** Search strategies for identifying potential investigational drugs.

| **Source** | **Purpose** |
| --- | --- |
| Company pipeline disclosures and corporate website | Identification of compounds and development status |
| ClinicalTrials.gov | Verification of development phase and ongoing studies |
| PubMed | Published evidence (including reviews and meta-analyses) |
| EMBASE | Published evidence (including reviews and meta-analyses) |
| Cochrane database (CENTRAL) | Published evidence (including reviews and meta-analyses) |
| Conference abstracts | Identification of emerging compounds and preliminary results |
| Google searches and grey literature | Retrieval of press releases and pipeline updates |
| Reference lists and manual searches | Additional cross-checking |

**Table 3S** - Earlier-generation anti-obesity pharmacotherapies with limited contemporary use

| Drug | Mechanism | Adm. | Route | Company | Clinical Phase | Authorization |
| --- | --- | --- | --- | --- | --- | --- |
| Phentermine/Topiramate | Centrally acting appetite suppressant + GABA modulation | OD | Oral | Vivus/Eisai | Approved | US |
| Bupropion/Naltrexone | Dopamine/norepinephrine reuptake inhibition + opioid receptor antagonism | BD | Oral | Currax | Approved | US, EU |
| Orlistat | Gastrointestinal lipase inhibitor | TID | Oral | Roche | Approved | US, EU |
| Setmelanotide | MC4 receptor agonist | OD | SC | Rhythm Pharmaceuticals | Approved | US, EU, UK |
| Mazindol | Sympathomimetic appetite suppressant | BD | Oral | Saniona | Approved | US, EU, UK |

Abbreviations: OD = once daily; BD = twice daily; TID = three times daily; SC = subcutaneous; MC4 = melanocortin-4.

**Table 4S** – Classes of drugs and individual obesity management medications approved for the treatment of obesity (as of April 1st, 2026).

| **Drug** | **Code** | **Mechanism** | **Adm.** | **Route** | **Company** | **Authorization** |
| --- | --- | --- | --- | --- | --- | --- |
| Semaglutide 2.4 mg | NN9535 | GLP-1 RA | OW | SC | Novo Nordisk | EU, US, UK, Japan, China, India, Australia |
| Liraglutide 3.0 mg | NN8022 | GLP-1 RA | OD | SC | Novo Nordisk | EU, US, UK, Japan, China, India, Australia |
| Oral semaglutide 50 mg | NN9924 | Oral GLP-1 RA | OD | Oral | Novo Nordisk | US |
| Semaglutide 7.2 mg | NN9535 | High-dose GLP-1 RA | OW | SC | Novo Nordisk | US, UK |
| Tirzepatide | LY3298176 | Dual GLP-1/GIP RA | OW | SC | Eli Lilly | EU, US, UK, Japan, China, India, Australia |
| Orforglipron | LY3502970 | Non-peptide GLP-1 RA | OD | Oral | Eli Lilly | US |
| Mazdutide | IBI362 | Dual GLP-1/Glucagon RA | OW | SC | Innovent/Eli Lilly | China |
| Ecnoglutide | XW003 | GLP-1 RA | OW | SC | Sciwind/Hansoh | China |
| Beinaglutide | BN-001 | GLP-1 RA | OD | SC | Benemae Pharmaceutical | China |

Abbreviations: OD = once daily; OW = once weekly; SC = subcutaneous; GLP-1: Glucagon-Like Peptide-1; GIP: Glucose-dependent Insulin Polipeptide; RA: Receptor Agonist;
